# Evolutionary algorithms and synthetic biology for directed evolution: commentary on “on the mapping of genotype to phenotype in evolutionary algorithms” by Peter A. Whigham, Grant Dick, and James Maclaurin

**DOI:** 10.1007/s10710-017-9292-1

**Published:** 2017-03-29

**Authors:** Douglas B. Kell

**Affiliations:** 1 School of Chemistry, The University of Manchester, 131, Princess St, Manchester, Lancs, M1 7DN UK; 2 The Manchester Institute of Biotechnology, The University of Manchester, 131, Princess St, Manchester, Lancs, M1 7DN UK; 3 Centre for Synthetic Biology of Fine and Speciality Chemicals, The University of Manchester, 131, Princess St, Manchester, Lancs, M1 7DN UK

**Keywords:** Directed evolution, Synthetic biology, Navigating search spaces, Intelligent operators

## Abstract

I rehearse two issues around the commentary of Whigham and colleagues. (1) There really are many more reasons than those given as to why natural evolution cannot reasonably find or select the ‘optimal’ individual. (2) A series of experimental molecular biology programmes, known generically as directed evolution, can use operators and selection schemes that natural evolution cannot. When developed further using the methods of synthetic biology, there are no operators or schemes for in silico evolution that cannot be applied precisely to directed evolution. The issues raised apply only to natural evolution but not to directed evolution.

## Introduction

The basic thesis of Whigham et al. [1] is that ‘molecular biology’, by which they actually mean ‘mechanisms of natural evolution’, is a relatively poor guide to the kinds of in silico optimisation problems that are typically the targets of evolutionary algorithms. While I have no disagreement with that high-level sentiment (indeed I have argued that biological genotype-phenotype mapping is entirely analogous to GP [2]!), they do omit some of the chief arguments as to why this is so. They also entirely disregard a very large literature using ‘molecular biology’ that is not ‘natural’ evolution but is aimed at producing (optimising) biological products via a set of processes typically referred to as ‘directed evolution’ (DE). These are therefore the points that I cover. A more extended overview of modern directed evolution may be found in [3].

## Why natural evolution is not necessarily ‘optimal’ in the sense of assuming that only the fittest survive

Whigham et al. [1] listed a series of reasons outlined by Dawkins [4] as to why this is so. Natural evolution is based on the processes of (i) diversity creation within a population (some or all of whom may interact and breed), (ii) ‘evaluation’ of fitness(es) and (iii) selection. Each of these may contribute to the fact that, even over an arbitrary time, populations do not converge such that they are populated only by individuals of the highest possible fitness for that (nominally unchanging) environment or objective function. Consequently there are many more reasons, at different levels of detail, as to why this is the case. Some include:

At level (i) (populations and diversity):

- Populations are normally not panmictic (where all can breed with all) so global optimality is then impossible.
- The size of the search space is so much greater than any population size that no test of optimality (in the NP complete sense) is possible; even 30mers of unmodified DNA have 10^18^ possible genotypes, which as 5 μm spots each would cover 29 km^2^ [5].
- When the population size is too small, some individuals who may yet contribute to optimality are simply squeezed out (especially in mutation-only organisms); this is known as Muller’s ratchet (e.g. [6–8]).
- Weak mutation and strong selection (see [9–14]) is common in many ecosystems, and traps individuals and populations in local minima (or maxima) from which neutral evolution cannot help them escape.
- Given that 3/64 codons are stop codons [15], the required mutation or recombination rates to explore the entire fitness landscape in a sensible time are incompatible with the sustenance of the population, given that most mutations are deleterious [16].

At level (ii) (fitnesses):

- The linkage between genotype, fitness and phenotype may often be very weak [17], for all kinds of reasons including epistasis [3] and the nonlinear properties of biochemical networks [18, 19].
- Non-genetic factors may intervene, e.g. while reproductive fitness is normally selected for, longevity may be preferred for social reasons (grandparents help with childcare, etc.); there is not just one fitness (hence optimality).

At level (iii) (selection)

- At the population level, while selection is based on the offspring more than the parents, simple stochastic events may blur the link between selection and fitness.
- For all kinds of reasons, selection is likely to (and does [20]) favour a differentiated population.

## Directed evolution (DE)

Directed evolution (a subset of closed loop optimisation [21]) refers to a set of biochemical procedures that are akin to natural evolution (in that they involve diversity creation and fitness-based selection). However, here the selection step is performed not by the organisms within their environment (as in natural evolution) but by an experimenter who has a specific goal, and thus selects individuals for further improvement based on those in a given generation. This is a glorified form of breeding (as in plant [22] and animal [23] breeding), but is typically done for improving individual proteins (e.g. as biocatalysts [24, 25]), where it is indeed sometimes known [17, 26, 27] as ‘molecular breeding’.

Classical methods of diversity generation used random in vitro methods of mutagenesis (e.g. [24, 28, 29]), or recombination (known here as ‘DNA shuffling’ [30, 31]), and selection was typically done on the basis of screening via a target biochemical assay (rather than true growth rate selection, e.g. as in [32, 33]).

Nowadays, the trend is to use the methods of synthetic biology, in which the experimenter has complete (or statistical) control over precisely which amino acid is expressed at which residue (e.g. [34]), literally by synthesising the encoding DNA, coupled to sophisticated in silico sequence optimisation methods (e.g. [35]). This synbio-based DE is formally equivalent to classical DE, but is typically cast in terms of a Design-Build-Test-Learn cycle [36–38]. Knowledge of gene sequences can be exploited directly [5, 39–41], and in some cases landscapes may be searched exhaustively [42]. What we learn from this (see also [43]) is that protein fitness landscapes are rugged but not pathologically so (e.g. [3, 44]), and that the evolutionary landscape metaphor can provide insights into both innovation [45] and science more generally [46].

This brings me nicely to my last point, elaborated in detail in Table 1 of [3], where we point out that many aspects of ‘classical’ and synthetic biology-based directed evolution differ massively from the same ones when they are compared to natural evolution, e.g. in terms of selection (pressure), mutation rates and their randomness, evolutionary ‘memory’, and the degree of epistasis allowed or encouraged. Even where these are solely quantitative differences, the effective emergent properties can make them appear to be qualitatively different. In particular, by controlling explicitly (at least statistically) the protein sequences in the population, we can combine the exploration and exploitation of fitness landscapes in DE by keeping low-fitness members in the population in a manner (arbitrarily long) that natural evolution would not be able to at any reasonable selection pressure. Notwithstanding, all these methods involve ‘molecular biology’.

In conclusion, if ‘molecular biology’ methods are applied to synbio-based directed evolution, any operator can be used, since any subsequent genetic sequence can be based arbitrarily on what has been seen at any time before, because it can be made de novo. These operators include very high mutation rates [16], three- (or more-)way matings, matings based on information gain and the Pareto front [47], complex crossover and mutation schemes such as ‘uniform’ crossover [48], explicit coevolutions based on sequence alignments and 3D structures (e.g. [49]), and so on. This, indeed, is precisely why the new methods of synthetic biology are so very powerful [3, 50]. Consequently, while recognising that natural evolution may be a rather poor guide to the utility of evolutionary algorithms in optimisation [1], we would stress the field of directed evolution, especially that based on synthetic biology, provides a highly productive domain for the cross-fertilisation of evolutionary algorithms and molecular biology.
